# Diagnosis of Respiratory Infections Using Syndromic Panels: A Ct-Based Approach Beyond Qualitative Detection

**DOI:** 10.3390/microorganisms14071450

**Published:** 2026-06-30

**Authors:** Maria Antonella Zingaropoli, Gianluca Bruno Tassone, Eleonora Coratti, Donatella Maria Rodio, Martina Bernassola, Roberta Campagna, Lucilla Caivano, Francesca Pulcinelli, Fabio Midulla, Gioacchino Galardo, Alessandra Pierangeli, Guido Antonelli, Ombretta Turriziani

**Affiliations:** 1 Microbiology and Virology Unit, Policlinico Umberto I, Sapienza University of Rome, 00161 Rome, Italy; d.rodio@policlinicoumberto1.it (D.M.R.); m.bernassola@policlinicoumberto1.it (M.B.); 2 Department of Molecular Medicine, Sapienza University of Rome, 00141 Rome, Italy; gianlucabruno.tassone@uniroma1.it (G.B.T.); eleonora.coratti@uniroma1.it (E.C.); roberta.campagna@uniroma1.it (R.C.); lucilla.caivano@uniroma1.it (L.C.); guido.antonelli@uniroma1.it (G.A.); ombretta.turriziani@uniroma1.it (O.T.); 3 Department of Pediatrics and Infantile Neuropsychiatry, Sapienza University of Rome, 00141 Rome, Italy; francesca.pulcinelli@uniroma1.it (F.P.); fabio.midulla@uniroma1.it (F.M.); 4 Department of Medical and Cardiovascular Sciences, Sapienza University of Rome, 00141 Rome, Italy; gioacchino.galardo@uniroma1.it; 5 Department of Well-being, Health and Environmental Sustainability (BeSSA), Sapienza University of Rome, 02100 Rieti, Italy; alessandra.pierangeli@uniroma1.it

**Keywords:** pediatric respiratory infection, adult respiratory infection, multiplex PCR, respiratory pathogen, respiratory virus, co-infection, Cycle threshold, diagnostic stewardship

## Abstract

This retrospective study, conducted between September 2023 and October 2025, evaluated age-related pathogen prevalence, seasonal dynamics, and Cycle threshold (Ct) values in nasopharyngeal specimens from hospitalized subjects with suspected acute respiratory infections (ARIs) using the multiplex syndromic panel. A median-based Delta Ct was calculated to define pathogen dominance in co-detections. A total of 2479 nasopharyngeal swabs were analyzed and 54.4% tested positive for at least one pathogen. Overall, the positivity rate and co-detections were found to be significantly higher in pediatric group than in the adult one (*p* < 0.0001 and *p* < 0.0001, respectively). Semi-quantitative analysis revealed that RSV and flu viruses maintained low Ct values irrespective of co-detection, whereas RV/EV, Adv, and human bocavirus (HBoV) exhibited significantly higher Ct values when co-detected. RV/EV showed higher Ct values versus human metapneumovirus A/B (*p* = 0.0014), human parainfluenza virus (*p* = 0.0007), flu virus (*p* = 0.0084) and RSV (*p* < 0.0001). Likewise, Adv demonstrated higher Ct values in comparison to RSV (*p* = 0.0046). Seasonal drivers typically dominate over persistent agents. Integrating semi-quantitative interpretation into syndromic panel reporting could enhance diagnostic stewardship, optimize antimicrobial use, and improve resource allocation in high-pressure clinical settings.

## 1. Introduction

The introduction of syndromic panels has revolutionized clinical microbiology by enabling the rapid, simultaneous identification of viruses, bacteria, fungi, and parasites. These tools are now widely integrated into routine clinical practice [1]. However, to maximize their clinical utility and economic sustainability, rigorous diagnostic stewardship measures are essential [2,3]. Inappropriate or excessive testing can impose a significant financial burden on healthcare systems with marginal benefits to patient management, particularly when results are difficult to interpret [4,5]. 

Unlike traditional methods, multiplex PCR panels offer high sensitivity for a broad range of pathogens. Despite these advantages, syndromic testing presents significant challenges, primarily regarding the interpretation of multiple positive targets. Moreover, because PCR detects nucleic acids regardless of organism viability, patients may remain positive long after clinical resolution. This persistence, coupled with the qualitative nature of most results, often obscures which pathogen is truly responsible for the patient’s symptoms, potentially leading to clinical ambiguity in cases of co-detection.

A promising approach to overcoming these interpretative challenges lies in the analysis of Cycle threshold (Ct) values. Although syndromic panels are primarily designed as qualitative tools, Ct values provide a semi-quantitative measure of viral load, offering significant insights into the replicative activity of the detected pathogens. Emerging evidence suggests that during co-infections, pathogens do not contribute equally to the clinical phenotype; instead, they often engage in complex interactions where one agent may emerge as the primary “driver” of the infection, while others act as clinically irrelevant pathogens, such as bystander or residual nucleic acids from previous episodes [6,7]. Mapping these pathogen-specific hierarchies is particularly vital in vulnerable populations, such as infants and elderly people, where the clinical management of multiple positive results can be controversial. Understanding which virus exhibits a significantly lower Ct value (indicating a higher viral load) in specific combinations––such as respiratory syncytial virus (RSV) versus rhinovirus/enterovirus (RV/EV) or primary pathogens versus persistent DNA viruses like human bocavirus (HBoV) and adenovirus (Adv)—could provide a biological basis for a more targeted diagnostic stewardship [8,9]. 

In this study, we analyzed two years of diagnostic results obtained at the Policlinico Umberto I Hospital in Rome, Italy, evaluating the prevalence and seasonal distribution of respiratory pathogens across different age groups in the post-pandemic era. Importantly, a median-based ΔCt analysis was employed to establish a hierarchy of viral dominance across co-detection patterns. This approach aims to provide a practical framework for the clinical interpretation of multiple positive targets, distinguishing acute infectious bursts from prolonged shedding to optimize isolation protocols and therapeutic interventions. 

## 2. Materials and Methods

### 2.1. Study Design and Sample Collection

A retrospective, single-center observational study was conducted at the Policlinico Umberto I (Rome, Italy), using data from September 2023 to October 2025. The study included hospitalized subjects with suspected acute respiratory infections (ARIs). Respiratory specimens were collected using nasopharyngeal flocked swabs and subsequently eluted in 1 mL of viral transport medium. Samples were processed immediately upon arrival at the laboratory.

### 2.2. Molecular Analysis: The QIAstat-Dx^®^ System

All specimens were analyzed using the QIAstat-Dx^®^ Respiratory SARS-CoV-2 Panel (Qiagen, Hilden, Germany), a fully automated multiplex real-time PCR platform. This system integrates nucleic acid extraction, amplification, and detection within a single-use disposable cartridge. The panel targets 23 pathogens: flu A (including H1N1 pdm09, H1, and H3 subtypes), flu B, severe acute respiratory syndrome coronavirus 2 (SARS-CoV-2), seasonal coronaviruses (229E, HKU1, NL63, OC43), human parainfluenza viruses 1, 2, 3, 4 (HPIV), RSV A/B, human metapneumovirus A/B (HMPV), Adv, HBoV, RV/EV (not differentiated), *Mycoplasma pneumoniae*, *Chlamydophila pneumoniae*, *Legionella pneumophila*, and *Bordetella pertussis*. All procedures were performed strictly following the manufacturer’s instructions.

### 2.3. Ct Value Extraction and Semi-Quantitative Analysis

Although the QIAstat-Dx is a qualitative platform, semi-quantitative data expressed as Ct values were retrieved for each positive target. Ct values were extracted through the QIAstat-Dx Operational Software (version 1.6.1), which allows for the visualization of individual amplification curves. These values were used as an indirect proxy for viral load, where lower Ct values correspond to higher nucleic acid concentrations. In cases of multiple detections, Ct values were compared to establish a hierarchy of dominance among co-detecting pathogens. Specifically, to compare two specific co-detected pathogens (e.g., Pathogen A and Pathogen B), a Delta Ct (ΔCt) was derived by subtracting the median Ct value of Pathogen B from the median Ct value of Pathogen A (ΔCt = median Ct Pathogen A − median Ct Pathogen B). A negative ΔCt value indicates that Pathogen A exhibits a lower median Ct than Pathogen B, defining Pathogen A as the “dominant” agent in this pairwise comparison. Conversely, a positive ΔCt indicates that Pathogen A has higher median Ct, defining it as “bystander” agent relative to Pathogen B. 

Although absolute Ct values cannot be directly compared across different pathogens due to target-specific primer/probe efficiencies, they provide a reliable semi-quantitative estimate of nucleic acid concentration for each specific target. For descriptive purposes, and in accordance with literature [7], Ct values were arbitrarily categorized into three tiers based on the assay’s analytical sensitivity and established literature: low Ct (<25, indicating high nucleic acid load), moderate Ct (25–30), and high Ct (>30, indicating low nucleic acid load). However, for all comparative statistical analyses, continuous Ct values were utilized.

### 2.4. Data Categorization and Statistical Analysis

The study population was stratified into two groups: pediatric (<18 years) and adult (≥18 years). The prevalence of each pathogen was then evaluated. Subsequent analysis was conducted to identify the most prevalent pathogen identified among the positive respiratory samples with multiple detections in relation to Ct values. Finally, the temporal distribution over time was determined by calculating the monthly positivity rate for each pathogen over the observed period. 

Statistical comparisons between groups (e.g., prevalence and co-detection rates) were performed using the Chi-square test. The non-parametric comparative Mann–Whitney U test was used for comparing the median Ct value of positive samples for both single and multiple targets. Furthermore, the non-parametric comparative Wilcoxon signed-rank test was used to compare the median Ct values of each pathogen with the median Ct values of the other pathogens detected in the same positive samples (paired data). Because Ct values inherently exhibit non-normal, often skewed distributions and are bounded by the assay’s limit of detection, they are highly susceptible to outliers. Consequently, for this type of semi-quantitative data, the median yields a more accurate representation of central tendency than the mean. A *p*-value of less than 0.05 was considered statistically significant (GraphPad Prism v.10).

## 3. Results

### 3.1. Seasonality of Respiratory Pathogens

This retrospective observational study analyzed respiratory samples collected over a 24-month period at Policlinico Umberto I (Rome, Italy) using a syndromic panel for upper respiratory tract infections. 

A total of 2479 respiratory specimens were collected from subjects with suspected ARIs. Of these, 1348 (54.4%) tested positive for at least one pathogen. Notably, the total number of detected pathogens exceeded the number of positive samples, reflecting the frequent occurrence of co-detections. Thus, a total of 1737 pathogens were detected, and RV/EV, RSV, and flu viruses were the most prevalent pathogens identified (Table 1, Supplementary Figure S1). 

**Figure 1:**
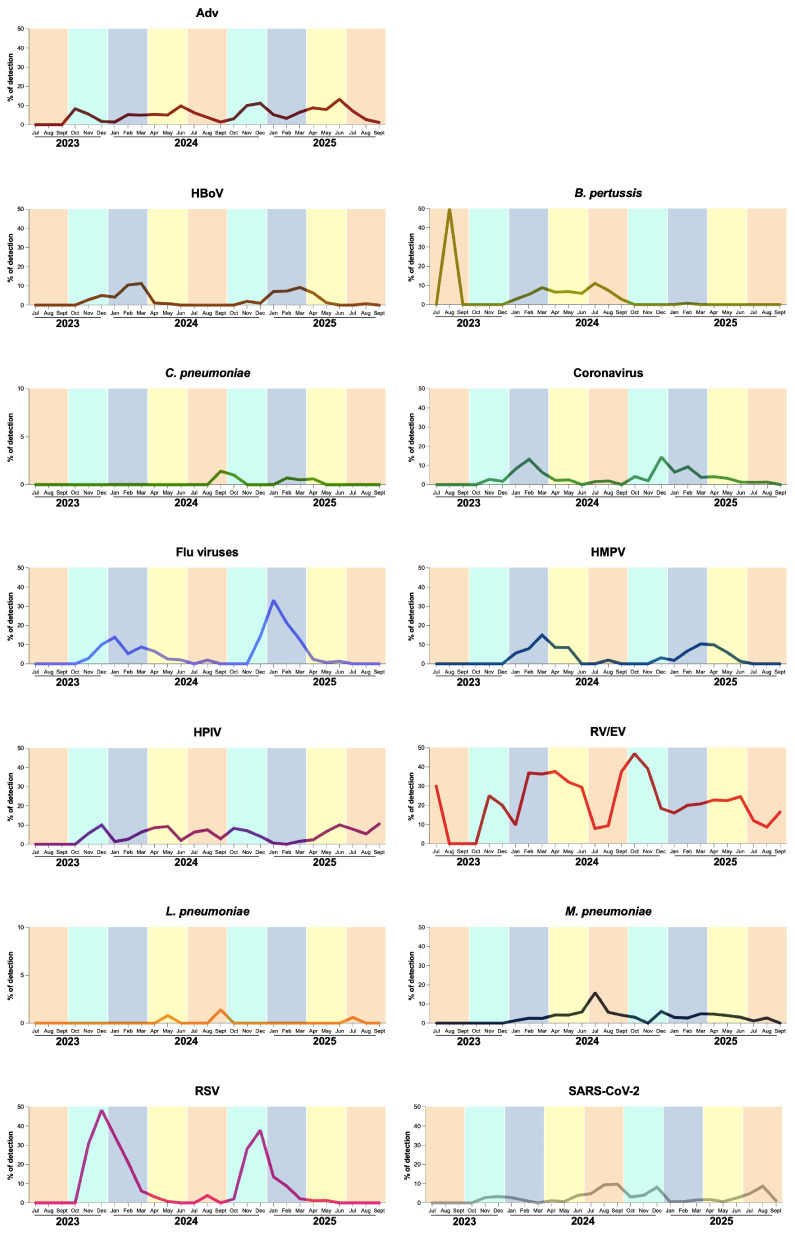
Evaluation of each pathogen in positive respiratory samples over time. Adv: adenovirus; HBoV: human bocavirus; HMPV: human metapneumovirus A/B; HPIV: human parainfluenza virus; RSV: respiratory syncytial virus A/B; RV/EV: rhinovirus/enterovirus; SARS-CoV-2: severe acute respiratory syndrome coronavirus 2.

The investigation of the seasonality of respiratory pathogens over a two-year observation period revealed distinct temporal patterns. RSV and flu viruses exhibited sharp, well-defined winter peaks, with RSV dominating the late autumn/early winter period, followed by a surge in flu virus cases (Figure 1). In contrast, RV/EV maintained a high and nearly constant prevalence throughout the study period (Figure 1). Similarly, Adv and HBoV showed lower but persistent detection rates across all seasons, without the marked fluctuations observed for primary seasonal viruses (Figure 1). Interestingly, *B. pertussis* and M. pneumoniae exhibited sporadic spikes. Specifically, a significantly higher percentage of *B. pertussis*-positive samples was detected in 2023/2024 compared to 2024/2025 (9.0 % versus 0.1%, *p* < 0.0001) (Figure 1).

**Figure 2:**
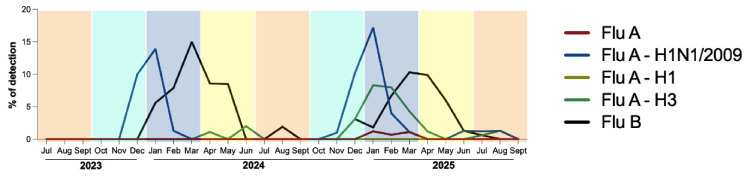
Evaluation of flu viruses in positive respiratory samples over time.

Regarding flu viruses, a significantly higher percentage of positive respiratory samples were observed in winter 2024/2025 compared to those observed in winter 2023/2024 (24.5% and 9.5%, respectively; *p* < 0.0001). Specifically, in contrast to flu A/H1N1, which was observed in both winters, a peak of flu A/H3 was exclusively detected during the winter of 2024/2025 (Figure 2). 

### 3.2. Distribution of Respiratory Pathogens in the Pediatric and the Adult Groups

The demographic profile and diagnostic outcomes of the study population, stratified also by age group, are detailed in Table 1. A significantly higher diagnostic yield was observed in the pediatric group compared to the adult one (69.8% and 28.4%, respectively; *p* < 0.0001). RV/EV was the most frequently identified agent overall, with a significantly higher detection rate in the pediatric group than in the adult one (*p* = 0.0181) (Table 1, Supplementary Figure S1A).

Other pathogens exhibited distinct age-related distribution patterns. Specifically, RSV and Adv showed a marked predominance in the pediatric group compared to adult one (*p* < 0.0001 and *p* < 0.0001, respectively) (Table 1, Supplementary Figure S1). HBoV followed a comparable trend, being rarely detected in the adult group (*p* < 0.0001) (Table 1, Supplementary Figure S1). In contrast, flu viruses were significantly more prevalent in the adult group than in the pediatric one (*p* < 0.0001), with flu A—H1N1/2009 and H3 subtypes being the predominant circulating strains (*p* < 0.0001 and *p* < 0.0001, respectively) (Table 1). Similarly, SARS-CoV-2 was detected more frequently in the adult group compared to the pediatric one (*p* = 0.0002) (Table 1, Supplementary Figure S1A). Among atypical bacteria, *M. pneumoniae* was significantly more prevalent in the pediatric group (*p* = 0.0333), whereas *L. pneumophila* was detected exclusively in the adult group (*p* = 0.0074) (Table 1, Supplementary Figure S1A).

### 3.3. Positive Respiratory Samples for Multiple Targets 

The complexity of pathogen co-detections is detailed in Table 2, which summarizes the distinct combination patterns identified across the study cohorts. Multiple detections were significantly more frequent in the pediatric group than in the adult group (28.6% and 8.8%, respectively; *p* < 0.0001) (Table 2).

Overall, RV/EV was the most prevalent pathogen in co-detections, followed by Adv and RSV (Table 2, Supplementary Figure S1B). As shown in Table 2, co-detections involving two pathogens were the most prevalent pattern. The combination of Adv + RV/EV was the most frequent, followed closely by RSV+RV/EV and HPIV+RV/EV. Notably, all Adv + RV/EV and RSV + RV/EV cases were identified exclusively in the pediatric group. Similarly, HMPV + RV/EV and HBoV + RV/EV combinations were detected almost entirely within the pediatric group (Table 2). Co-detections involving three pathogens were also documented almost exclusively in the pediatric group. Various triple combinations were recorded, with Adv + RSV + RV/EV and HBoV + HPIV + RV/EV being the most prevalent. Triple detections involving *B. pertussis* or *M. pneumoniae* were also observed, albeit less frequently. Finally, although rare, three distinct quadruple detection patterns were identified, all in the pediatric groups (Table 2). 

### 3.4. Ct Value Analysis

**Figure 3:**
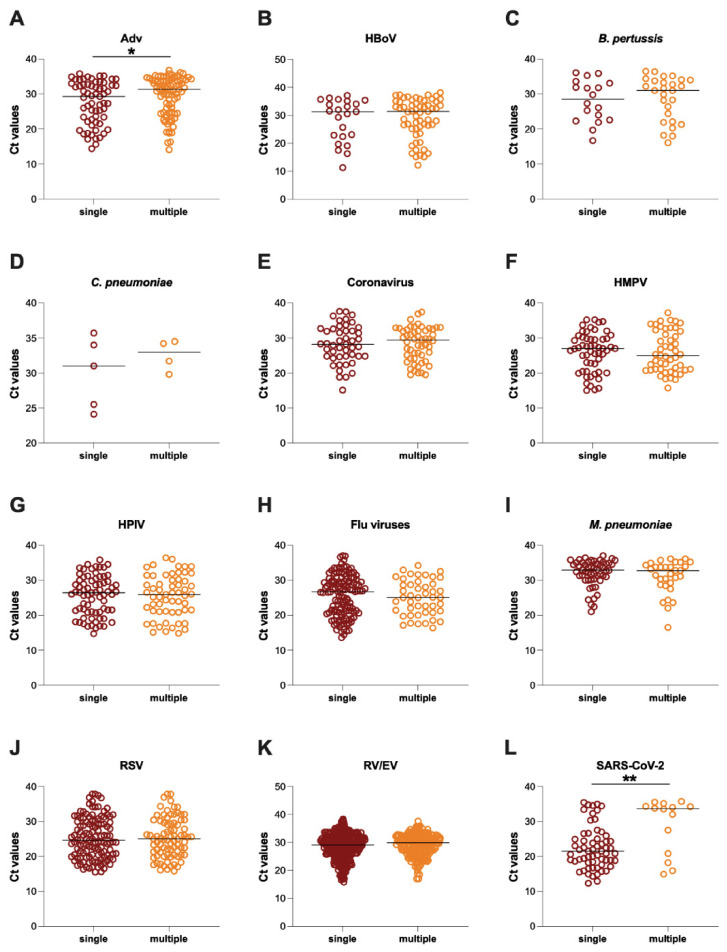
Evaluation of Ct values for each pathogen in positive samples for single and multiple targets. The non-parametric comparative Mann–Whitney test was used for comparing the median Ct value of positive samples for both single and multiple targets. The median values and interquartile ranges are documented in detail in Supplementary Table S1. Adv: adenovirus; HBoV: human bocavirus; HMPV: human metapneumovirus A/B; HPIV: human parainfluenza virus; RSV: respiratory syncytial virus A/B; RV/EV: rhinovirus/enterovirus; SARS-CoV-2: severe acute respiratory syndrome coronavirus 2. *: *p* < 0.05, **: *p* < 0.01.

To investigate the possible interactions between co-detecting pathogens, Ct values were analyzed, comparing single-target with multiple-target detections for each pathogen (Figure 3, Supplementary Table S1). For most high-impact respiratory pathogens, including RSV, flu viruses, HPIV, and HMPV (Figure 3J, 3H, 3G and 3F, respectively), Ct values demonstrated remarkable stability, with median values in the moderate range (25–30 Ct) irrespective of whether the pathogen was detected alone or in combination with others (Supplementary Table S1). This suggests that these pathogens maintain consistent replication dynamics regardless of co-infection status. Both HBoV and RV/EV exhibited distinct patterns. HBoV showed consistently high Ct values in both single and multiple detections (median Ct 31.3 and 31.4, respectively; Figure 3B), suggestive of persistent colonization rather than acute infection. In contrast, RV/EV maintained moderate Ct values in both single and multiple detections (median Ct value 24.6 and 25.0; Figure 3K, respectively, Supplementary Table S1).

Statistically significant differences in Ct values between single and multiple detections were observed for two pathogens. For Adv, Ct values were significantly higher in samples positive for multiple targets compared to those observed for Adv alone (31.4 [24.7–33.9] and 29.3 [22.1–32.7], respectively; *p* = 0.0392), corresponding to an approximately 4.3-fold lower viral load in co-detections (Figure 3A, Supplementary Table S1). A similar but more pronounced pattern was observed for SARS-CoV-2, where markedly higher Ct values were recorded in multiple-target positive samples compared to single-target positive samples (33.7 [20.2–34.5] and 21.5 [18.3–25.5], respectively; *p* = 0.0071), corresponding to an approximately 4600-fold lower viral load in co-detections (Figure 3L, Supplementary Table S1). This dramatic shift suggests that when SARS-CoV-2 is co-detected with other respiratory pathogens, it is more likely to represent residual nucleic acid from a previous infection rather than an active, high-replication infection.

### 3.5. Head-to-Head Ct Value Comparison in Sample Positive for Multiple Targets 

Given the high frequency of detection of RV/EV, Adv and RSV across all age groups and their frequent presence in co-detections, a comparative Ct value analysis was employed to differentiate dominant pathogens from potential “bystanders”. This analysis was conducted separately for dual detections and for samples in which three or more pathogens were detected.

**Figure 4:**
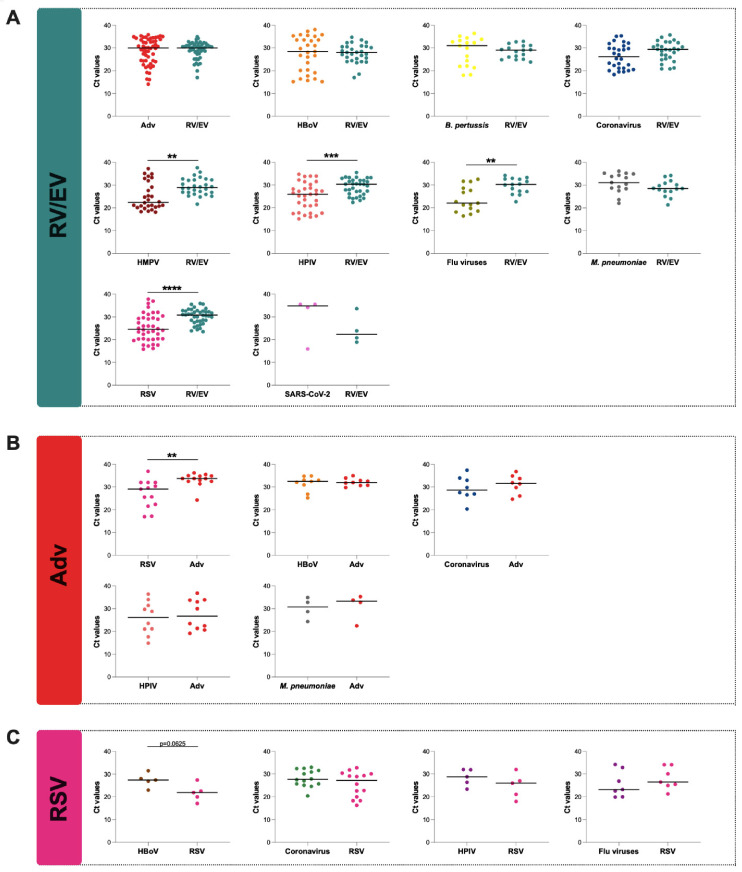
Comparison of Ct values of RV/EV (**A**), Adv (**B**), and RSV (**C**) in positive samples for multiple targets. The non-parametric comparative Wilcoxon test was used for comparing median Ct values of RV/EV, Adv, RSV and the other pathogen in positive samples that were for multiple targets. The median values and interquartile ranges are documented in detail in Supplementary Table S2. Adv: adenovirus; HBoV: human bocavirus; HMPV: human metapneumovirus A/B; HPIV: human parainfluenza virus; RSV: respiratory syncytial virus A/B; RV/EV: rhinovirus/enterovirus; SARS-CoV-2: severe acute respiratory syndrome coronavirus 2. **: *p* < 0.01, ***: *p* < 0.001, ****: *p* < 0.0001.

As shown in Figure 4A, RV/EV exhibited significantly higher Ct values when co-detected with HMPV (*p* = 0.0014), HPIV (*p* = 0.0007), flu viruses (*p* = 0.0084) and RSV (*p* < 0.0001). In contrast, no significant differences in Ct values were found when RV/EV was co-detected with Adv, HBoV, or *B. pertussis* (Figure 4A, Supplementary Table S2). This pattern was consistent in both co-detection for two pathogens and in samples positive for three or more pathogens (Supplementary Figure S2).

For Adv, Ct values were generally comparable to those of co-detected pathogens across most combination patterns. However, a notable exception was observed in the RSV + Adv combination, where Adv exhibited significantly higher Ct values compared to RSV (*p* = 0.0046). This difference exhibited a comparable trend in co-detection samples that were positive for two pathogens, although this did not reach statistical significance, potentially due to the limited sample size. Conversely, the difference attained statistical significance in samples that were positive for three or more pathogens (Supplementary Figure S2).

Conversely, RSV demonstrated significantly lower Ct values when co-detected with HBoV, in samples positive for two or more pathogens (Figure 4C, Supplementary Table S2). A similar trend toward lower Ct values for RSV was observed with co-detection of Coronavirus in samples positive for two pathogens, although median Ct values were comparable in samples positive for three or more pathogens (Figure 4C, Supplementary Figure S2). 

In all other combinations, including those with HPIV and flu viruses, RSV exhibited comparable or lower Ct values, although some pairings did not reach statistical significance, likely due to reduced sample sizes (Figure 4C, Supplementary Table S2). 

### 3.6. Hierarchical Matrix of Pathogenic Dominance

**Figure 5:**
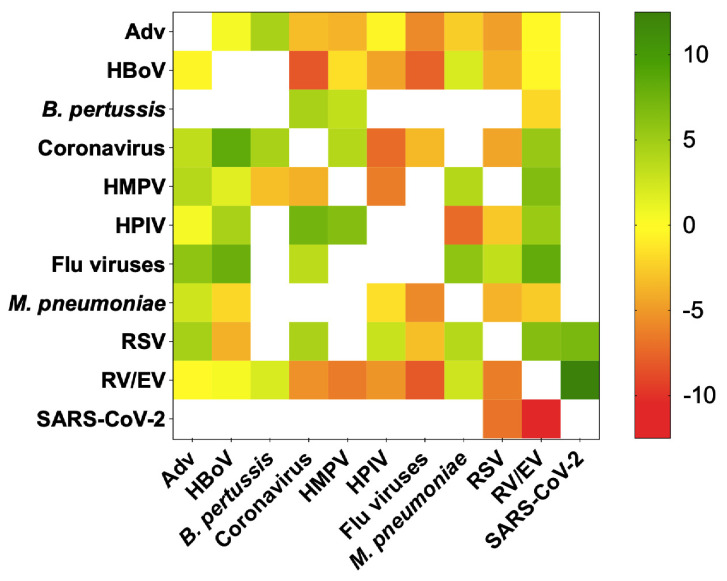
Hierarchical Mapping of Pathogen Dominance. The heatmap displays pairwise comparisons of pathogens detected in co-detections. Red/orange intensity indicates that the pathogen on the X-axis has lower Ct values (i.e., a higher viral load, negative ΔCt) than the corresponding pathogen on the Y-axis. Green intensity indicates that the pathogen on the X-axis has higher Ct values (i.e., lower viral load, positive ΔCt) than Ct values of the pathogen on the Y-axis. Yellow cells indicate comparable Ct values. Blank cells indicate pathogen combination not observed. Adv: adenovirus; HBoV: human bocavirus; HMPV: human metapneumovirus A/B; HPIV: human parainfluenza virus; RSV: respiratory syncytial virus A/B; RV/EV: rhinovirus/enterovirus; SARS-CoV-2: severe acute respiratory syndrome coronavirus 2.

To provide a comprehensive overview of the interactions between co-detected pathogens, a heatmap was constructed based on median ΔCt values (Figure 5). Combinations with n ≤ 2 were excluded to ensure statistical robustness. 

In Figure 5, which considers all co-detections (two, three or four pathogens), green indicates positive ΔCt values (higher Ct relative to co-pathogen, suggesting bystander status), while red indicates negative ΔCt values (lower Ct, suggesting dominant status). Adv, HBoV, RV/EV, and SARS-CoV-2 exhibited predominantly green profiles when matched against major respiratory pathogens (e.g., HMPV, HPIV, flu viruses, RSV), confirming their tendency to act as bystander pathogens in most co-detections. Conversely, RSV and flu viruses displayed consistently red/orange profiles, demonstrating significantly lower Ct values even when co-detected with atypical bacteria or other viruses, particularly Adv, HBoV, and RV/EV (Figure 5). Intermediate patterns (orange/yellow) were observed for pathogens such as coronavirus, indicating comparable Ct values to their co-detected partners and suggesting a more variable role depending on the specific combination. The same assessment was performed considering only samples positive for two pathogens (Supplementary Figure S3). The hierarchical patterns remained consistent with those observed in all combination patterns, with RSV and flu viruses maintaining their dominant role, while RV/EV, Adv, and HBoV consistently exhibited subordinate patterns (Supplementary Figure S3). Notably, this consistency across different infection complexities confirms the robustness of the ΔCt-based approach.

## 4. Discussion

While the widespread implementation of syndromic panels has revolutionized clinical microbiology, the interpretation of multiple positive targets continues to pose a significant clinical challenge. This 24-month observational study analyzed respiratory specimens from pediatric and adult subjects to evaluate pathogen prevalence and semi-quantitative Ct values, aiming to distinguish dominant pathogens from “bystanders”.

Consistent with literature, positivity rates were significantly higher in the pediatric group [10]. RV/EV was the most prevalent pathogen year-round, whereas RSV and flu viruses exhibited sharp winter peaks, aligning with pre-pandemic seasonal patterns [11]. Notably, we observed a dramatic *B. pertussis* epidemic spike in 2023/2024, likely reflecting cyclical epidemiology amplified by pandemic-related vaccination gaps [12]. Furthermore, flu A/H3 dominance in winter 2024/2025 correlated with higher detection rates in adults, aligning with known severity patterns [13]. 

Age-specific bacterial patterns emerged: *L. pneumophila* was exclusive to adult group, reflecting known risk factors, while *M. pneumoniae* predominated in children [14,15]. 

Co-infections were significantly more frequent in the pediatric group, attributed to immunological naivety and social exposure [16,17,18,19,20,21].

The Ct analysis revealed a clear replicative hierarchy. RSV and flu viruses acted as “apex pathogens”, maintaining low Ct values (high load) regardless of co-detection, suggesting high replicative fitness and a primary driver role [22,23]. Conversely, RV/EV, Adv, and HBoV frequently exhibited moderate or high Ct values in co-detections, supporting a bystander role likely due to prolonged shedding rather than acute replication [24,25,26,27]. This concept is particularly relevant for atypical bacteria such as *C. pneumoniae* and *M. pneumoniae*, which are well-documented to persist as asymptomatic carriers in the respiratory tract. Therefore, their mere qualitative detection in a syndromic panel does not always imply an active, symptomatic infection, but may simply reflect a carrier state. Specifically, Adv and SARS-CoV-2 showed significantly higher Ct values in multiple-target samples, potentially indicating a bystander role or late-phase shedding [28,29,30,31,32,33]. 

Our findings align with and extend previous studies on viral interactions in co-infections. Laurie et al. [34] demonstrated in a ferret model that viral interference between influenza strains is dependent on both the interval between infections and a viral hierarchy, where certain strains consistently dominate others. This viral hierarchy concept is further supported by computational models based on the competitive exclusion principle, which suggest that when two viruses compete for the same ecological niche, the fitter strain may out-compete and eliminate the rival strain [35]. 

Moreover, recent experimental work by Cheemarla et al. [36] investigating SARS-CoV-2 and influenza A interactions in human airway epithelium demonstrated that influenza A/H3N2 strongly interfered with SARS-CoV-2 replication through robust interferon (IFN) production, while SARS-CoV-2 induced only marginal IFN responses and could suppress IFN production during coinfections.

The hierarchical heatmap confirmed these dynamics: RSV demonstrated a consistent pattern of superior performance in comparison to RV/EV and Adv. Concurrently, perennial pathogens exhibited a marginal presence within the designed “green zone” of the heatmap results, when evaluated in conjunction with seasonal drivers [37]. Beyond dual detections, triple and quadruple combinations were documented almost exclusively in the pediatric group, raising questions about the cumulative pathogenic burden in this population [38]. The consistency of pathogen hierarchies across both dual and multiple detection scenarios strengthens the validity of the present ΔCt-based framework. This finding extends current knowledge by suggesting that pathogen dominance patterns remain consistent in scenarios involving multiple detections, which are common in pediatric populations [38].

These findings support the hypothesis that not all detected pathogens contribute equally to symptomatic burden. This distinction is particularly relevant in the current clinical landscape, where the high sensitivity of multiplex panels can inadvertently encourage over-testing and lead clinicians to over-trust these assays as a definitive diagnostic modality. The mere qualitative detection of a nucleic acid target—especially for pathogens prone to prolonged shedding—does not necessarily equate to active clinical disease. Relying solely on a positive/negative output without integrating semi-quantitative data (Ct values) or the patient’s clinical context can result in the misinterpretation of bystander pathogens as primary drivers, potentially leading to unnecessary treatments. 

Despite the insights of the present study, several limitations must be acknowledged. First, the retrospective, single-center design may introduce selection bias and limit the generalizability of our findings. Second, the interpretation of Ct values as indicators of pathogen “dominance” must be made cautiously. Ct values can vary depending on assay performance, sample quality, and amplification efficiency, and are not strictly equivalent to standardized quantitative nucleic acid load measurements. Third, due to the laboratory-based nature of this retrospective study, we lacked comprehensive clinical data to directly confirm whether the pathogen with the lowest Ct value was the true primary causative agent. Similarly, we were unable to adjust for potential confounding factors such as underlying diseases, immune status, vaccination history, prior antimicrobial use, or duration from symptom onset to sampling, all of which could influence Ct values and pathogen detection. Moreover, some pathogen combinations, particularly triple and quadruple co-detections, had very small sample sizes, which may weaken the statistical reliability of the subgroup analyses and heatmap interpretation for these specific rare combinations. Finally, the observed pathogen hierarchies were predominantly derived from pediatric co-detections, as multiple detections were significantly less frequent in adults. This pediatric bias limits the generalizability of our findings to the broader respiratory syndromic panel context across all age groups. Future prospective studies integrating detailed clinical metadata and standardized viral load quantification are warranted to validate the proposed ΔCt hierarchies.

## 5. Conclusions

In conclusion, understanding the pathogen etiology and the age-specific incidence is vital to provide modern management of patients with infectious diseases and to optimize public health strategies. Multiplex syndromic panels are undeniably valuable tools that allow for rapid and prompt diagnosis and significantly increase detection rates. However, their purely qualitative nature can sometimes mask or hinder the identification of the true causative agent in co-infections. Leveraging Ct values has the potential to guide clinicians in distinguishing primary drivers from irrelevant bystanders, particularly during winter surges. Whilst prospective validation studies are still required to confirm that this approach directly improves antimicrobial stewardship and clinical outcomes in practice, we advocate for the integration of semi-quantitative reporting, as it provides crucial insights that consolidate the need for robust diagnostic stewardship. 

## Figures and Tables

**Table 1:** Pathogens detected by the QIAstat-Dx^®^ Respiratory SARS-CoV-2 Panel, regardless of single or multiple identifications.

	Overall, n (%)	Pediatric, n (%)	Adult, n (%)	*p* Values
**females/males**	1086/1393	697/857	389/536	ns
**age, years (IQR)**	7.0 (0.9–59.9)	1.4 (0.3–5.8)	69.6 (53.8–79.8)	-
**positive/negative samples**	1348/1129	1085/469	263/662	*p* < 0.0001
**n° detected pathogens**	1737	1450	287	-
**n° sample positive for single target**	1014	774	240	-
**n° sample positive for multiple target**	333	310	23	*p* < 0.0001
**virus + virus**	272	254	19	-
**virus + bacterium**	60	56	4	-
**bacterium + bacterium**	1	1	0	-
**Pathogens**				
** ** **RV/EV**	569 (42.2)	475 (43.8)	94 (35.7)	*p* = 0.0181
** ** **RSV**	203 (15.1)	197 (18.2)	6 (2.3)	*p* < 0.0001
** ** **Flu viruses**	178 (13.2)	114 (10.5)	64 (24.3)	*p* < 0.0001
** ** **Flu A**	5 (0.4)	4 (0.4)	1 (0.4)	ns
** ** **Flu A—H1N1/2009**	71 (5.3)	39 (3.6)	32 (12.2)	*p* < 0.0001
** ** **Flu A—H1**	-	-	-	-
** ** **Flu A—H3**	43 (3.2)	18 (1.7)	25 (9.5)	*p* < 0.0001
** ** **Flu B**	60 (4.5)	56 (5.2)	4 (1.5)	*p* = 0.0073
** ** **Adv**	151 (11.2)	147 (13.5)	4 (1.5)	*p* < 0.0001
** ** **HPIV**	131 (9.7)	102 (9.4)	28 (10.6)	ns
** ** **1**	8 (0.6)	8 (0.7)	0 (0.0)	ns
** ** **2**	17 (1.3)	17 (1.6)	0 (0.0)	ns
** ** **3**	74 (5.5)	55 (5.1)	19 (7.2)	ns
** ** **4**	32 (2.4)	22 (2.0)	10 (3.8)	ns
** ** **HMPV**	104 (7.7)	88 (8.1)	16 (6.1)	ns
** ** **Coronavirus**	100 (7.4)	74 (6.8)	26 (9.9)	ns
** ** **229E**	4 (0.3)	2 (0.2)	2 (0.8)	ns
** ** **HKU1**	22 (1.6)	14 (1.3)	8 (3.0)	ns
** ** **NL63**	21 (1.6)	20 (1.8)	1 (0.4)	ns
** ** **OC43**	54 (4.0)	39 (3.6)	15 (5.7)	ns
** ** ***M. pneumoniae***	85 (6.3)	76 (7.0)	9 (3.4)	*p* = 0.0333
** ** **HBoV**	82 (6.1)	80 (7.4)	2 (0.8)	*p* < 0.0001
** ** **SARS-CoV-2**	75 (5.6)	47 (4.3)	28 (10.6)	*p* = 0.0002
** ** ***B. pertussis***	45 (3.3)	42 (3.9)	3 (1.1)	*p* = 0.0220
** ** ***C. pneumoniae***	9 (0.7)	7 (0.6)	2 (0.8)	ns
** ** ***L. pneumoniae***	3 (0.2)	0 (0.0)	3 (1.1)	*p* = 0.0074

Percentages refer to total number of positive respiratory samples (overall n = 1348, pediatric group n = 1085, adult group n = 263). The non-parametric comparative Mann–Whitney test was used for comparing median values of pediatric and adult group. RV/EV: rhinovirus/enterovirus; RSV: respiratory syncytial virus A/B; Adv: adenovirus; HPIV: human parainfluenza virus; HMPV: human metapneumovirus A/B; HBoV: human bocavirus; SARS-CoV-2: severe acute respiratory syndrome coronavirus 2; ns: not significant (*p* > 0.05).

**Table 2:** The respiratory combination pattern in both the pediatric and adult groups.

Respiratory Combination Patterns	Overall	Pediatric	Adult
**Two pathogens**			
Adv + HBoV	5	5	0
Adv + *B. pertussis*	1	1	0
Adv + *C. pneumoniae*	1	1	0
Adv + coronavirus	3	3	0
Adv + HMPV	4	4	0
Adv + HPIV	4	4	0
Adv + flu viruses	2	2	0
Adv + *M. pneumoniae*	1	1	0
Adv + RSV	5	5	0
Adv + RV/EV	33	33	0
Adv + SARS-CoV-2	1	1	0
HBoV + coronavirus	4	3	1
HBoV + HPIV	1	1	0
HBoV + HMPV	1	1	0
HBoV + flu viruses	7	7	0
HBoV + *M. pneumoniae*	4	4	0
HBoV + RSV	4	4	0
HBoV + RV/EV	16	15	1
*B. pertussis* + coronavirus	1	1	0
*B. pertussis* + HPIV	1	1	0
*B. pertussis* + HMPV	3	3	0
*B. pertussis* + flu viruses	2	1	1
*B. pertussis* + *M. pneumoniae*	1	1	0
*B. pertussis* + RV/EV	14	13	1
*B. pertussis* + SARS-CoV-2	1	1	0
*C. pneumoniae* + flu viruses	1	1	0
Coronavirus + HPIV	4	2	2
Coronavirus + HMPV	3	3	0
Coronavirus + flu viruses	6	3	3
Coronavirus + RSV	8	8	0
Coronavirus + RV/EV	5	5	0
HPIV + HMPV	2	2	0
HPIV + HPIV *	1	0	1
HPIV + *M. pneumoniae*	2	2	0
HPIV + RSV	4	4	0
HPIV + RV/EV	25	24	1
HPIV + SARS-CoV-2	2	2	0
HMPV + flu viruses	2	2	0
HMPV + RSV	2	2	0
HMPV + RV/EV	19	17	2
Flu viruses + *M. pneumoniae*	3	2	1
Flu viruses + RSV	6	5	1
Flu viruses + RV/EV	10	6	4
*M. pneumoniae* + RSV	4	4	0
*M. pneumoniae* + RV/EV	11	10	1
RSV + RV/EV	30	30	0
RSV + SARS-CoV-2	6	6	0
RV/EV + SARS-CoV-2	4	2	2
**Three pathogens**			
Adv + HBoV + flu viruses	1	1	0
Adv + HBoV + HMPV	1	1	0
Adv + HBoV + RV/EV	2	2	0
Adv + *C. pneumoniae* + RV/EV	1	1	0
Adv + coronavirus + HPIV	1	1	0
Adv + coronavirus + RSV	1	1	0
Adv + coronavirus + RV/EV	2	2	0
Adv + HPIV + *M. pneumoniae*	1	1	0
Adv + HPIV + HMPV	1	1	0
Adv + HPIV + RSV	1	1	0
Adv + HPIV + RV/EV	1	1	0
Adv + HMPV + RV/EV	3	3	0
Adv + flu viruses + RV/EV	1	1	0
Adv + *M. pneumoniae* + RV/EV	2	2	0
Adv + RSV + RV/EV	5	5	0
HBoV + coronavirus + HMPV	2	2	0
HBoV + coronavirus + RV/EV	3	3	0
HBoV + HMPV + RV/EV	1	1	0
HBoV + HPIV + RV/EV	4	4	0
HBoV + flu viruses + RV/EV	2	2	0
HBoV + RSV + RV/EV	1	1	0
*B. pertussis* + coronavirus + RV/EV	2	2	0
*B. pertussis* + HMPV + RV/EV	1	1	0
*C. pneumoniae* + flu viruses + RV/EV	1	1	0
Coronavirus + flu viruses + RSV	1	0	1
Coronavirus + HMPV + RV/EV	1	1	0
Coronavirus + *M. pneumoniae* + RV/EV	1	1	0
Coronavirus + RSV + RV/EV	3	3	0
HMPV + flu viruses + RV/EV	1	1	0
HMPV + HPIV + RV/EV	1	1	0
HMPV + *M. pneumoniae* + RV/EV	1	1	0
Four pathogens			
Adv + coronavirus + coronavirus + RV/EV	1	1	0
Adv + coronavirus + RSV + RV/EV	1	1	0
Adv + HMPV + HPIV + RV/EV	1	1	0

*: HPIV-3 + HPIV-4. Adv: adenovirus; HBoV: human bocavirus; HMPV: human metapneumovirus A/B; HPIV: human parainfluenza virus; RSV: respiratory syncytial virus A/B; RV/EV: rhinovirus/enterovirus; SARS-CoV-2: severe acute respiratory syndrome coronavirus 2.

## Data Availability

The original contributions presented in this study are included in the article/Supplementary Materials. Further inquiries can be directed to the corresponding author.

## Supplementary Materials

The following supporting information can be downloaded at: https://www.mdpi.com/article/10.3390/microorganisms14071450/s1, Figure S1: The percentages of each pathogen’s identification, among the total of respiratory samples in each separate pediatric and adult group (A). The percentages of each pathogen in positive samples for multiple targets (B). Adv: Adenovirus; HBoV: Human Bocavirus; HMPV: Human metapneumovirus A/B; HPIV: Human parainfluenza virus; RSV: Respiratory syncytial virus A/B; RV/EV: Rhinovirus/Enterovirus; Figure S2: Comparison of Ct values of RV/EV (A), Adv (B), and RSV (C) in positive samples only for two targets; Figure S3: Hierarchical Mapping of Pathogen Dominance in sample positive for two pathogens; Table S1: Comparison of Cycle threshold (Ct) values between single and multiple pathogen detections; Table S2: Cycle threshold (Ct) values of each pathogen in respiratory combination patterns.

## Abbreviations

The following abbreviations are used in this manuscript:

Ct	Cycle threshold
RSV	respiratory syncytial virus
RV/EV	rhinovirus/enterovirus
HBoV	human bocavirus
Adv	adenovirus
ARIs	acute respiratory infections
SARS-CoV-2	severe acute respiratory syndrome coronavirus 2
HMPV	human metapneumovirus A/B
ΔCt	Delta Ct values
