# Supplementary material for: Diagnosis of Respiratory Infections Using Syndromic Panels: A Ct-Based Approach Beyond Qualitative Detection
Source: Microorganisms. 2026 Jun 30;14(7):1450. doi: 10.3390/microorganisms14071450 (PMC13413469; doi:10.3390/microorganisms14071450)
Supplement: Supplementary file 1 [file microorganisms-14-01450-s001.zip › microorganisms-4311932-supplementary.pdf]

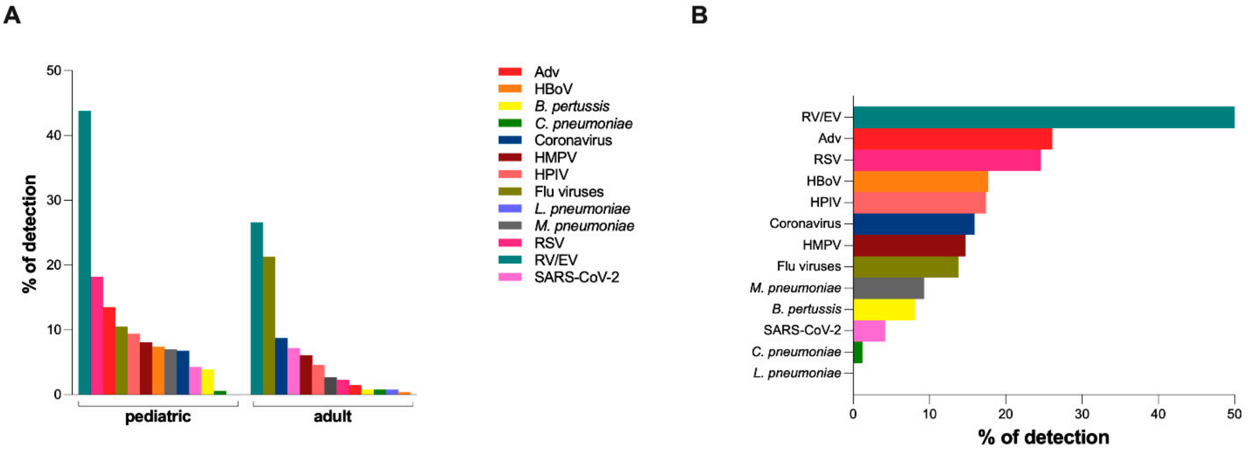

Supplementary Figure S1. The percentages of each pathogen's identification, among the total of respiratory samples in each separate pediatric and adult group (A). The percentages of each pathogen in positive samples for multiple targets (B). Adv: Adenovirus; HBoV: Human Bocavirus; HMPV: Human metapneumovirus A/B; HPIV: Human parainfluenza virus; RSV: Respiratory syncytial virus A/B; RV/EV: Rhinovirus/Enterovirus.

	single	multiple	p values
**Pathogens**			
**Adv**	29.3 (22.1-32.7), n=63	31.4 (24.7-33.9), n=87	p=0.0392
**HBoV**	31.3 (22.4-35.1), n=23	31.4 (25.3-34.9), n=59	ns
B. pertussis	28.6 (22.5-33.2), n=18	31.0 (22.1-34.0), n=27	ns
C. pneumoniae	31.0 (24.8-34.9), n=5	33.0 (30.3-34.4), n=4	ns
**Coronavirus**	28.2 (24.8-32.6), n=47	29.4 (24.1-32.4), n=54	ns
**HMPV**	26.4 (20.9-30.0), n=72	26.0 (21.1-30.5), n=58	ns
**HPIV**	27.0 (20.4-29.8), n=55	25.0 (21.0-32.1), n=49	ns
**Flu viruses**	26.7 (20.9-29.8), n=132	25.0 (20.8-29.1), n=46	ns
L. pneumoniae	31.5 (18.2-34.8), n=3	na, n=0	ns
M. pneumoniae	32.9 (30.3-34.9), n=54	32.8 (28.7-34.3), n=31	ns
**RSV**	29.1 (25.8-31.8), n=359	29.9 (26.3-32.0), n=210	ns
**RV/EV**	24.6 (20.4-29.9), n=121	25.0 (20.4-29.7), n=82	ns
**SARS-CoV-2**	21.5 (18.3-25.5), n=61	33.7 (20.2-34.5), n=14	p=0.0071

Supplementary Table S1. Comparison of Cycle threshold (Ct) values between single and multiple pathogen detections. Data are expressed as median (interquartile range, IQR). The number of positive samples (n) is reported for each category. Statistical significance was determined using the Mann-Whitney U test.

Adv: Adenovirus; HBoV: Human Bocavirus; HMPV: Human metapneumovirus A/B; HPIV: Human parainfluenza virus; RSV: Respiratory syncytial virus A/B; RV/EV: Rhinovirus/Enterovirus; SARS-CoV-2: Severe acute respiratory syndrome coronavirus 2; na: not available; ns: not significant (p>0.05).

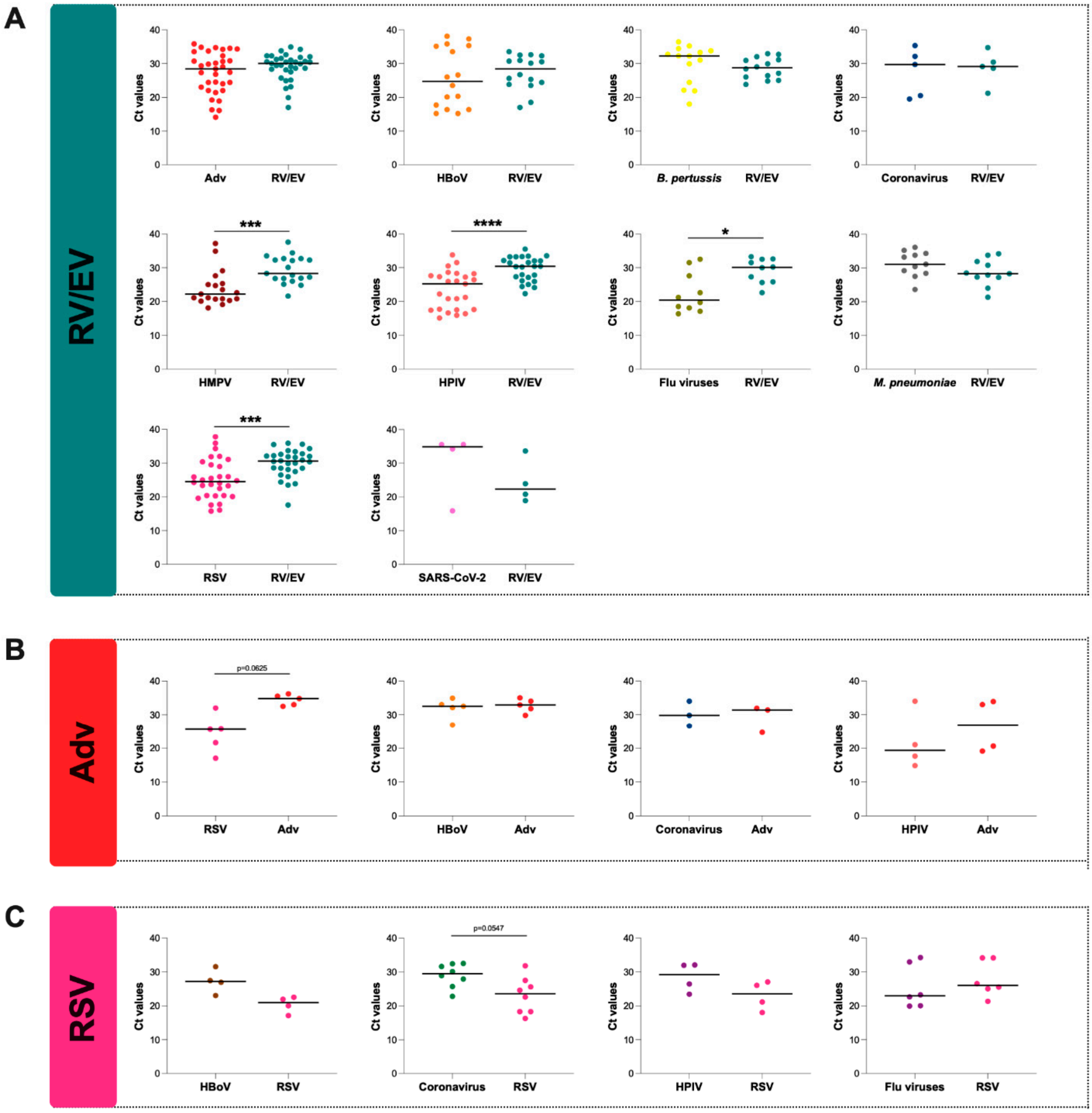

Supplementary Figure S2. Comparison of Ct values of RV/EV (A), Adv (B), and RSV (C) in positive samples only for two targets. The non-parametric comparative Wilcoxon test was used for comparing median Ct values of RV/EV, Adv, RSV and the other pathogen in positive samples that were for two targets. Adv: Adenovirus; HBoV: Human Bocavirus; HMPV: Human metapneumovirus A/B; HPIV: Human parainfluenza virus; RSV: Respiratory syncytial virus A/B; RV/EV: Rhinovirus/Enterovirus; SARS-CoV-2: Severe acute respiratory syndrome coronavirus 2.

	Ct values	p value
**Adv *vs* HBoV, n=9**	32.0 (30.8-35.5) *vs* 32.5 (29.0-34.0)	ns
**Adv *vs* *B. pertussis*, n=1**	22 (22.-2-2.22) *vs* 36.5 (36.5-36-5)	ns
**Adv *vs* *C. pneumoniae*, n=2**	35.0 (34.7-35.2) *vs* 34.4 (34.2-34.5)	ns
**Adv *vs* Coronavirus, n=10**	31.7 (28.9-34.1) *vs* 28.4 (25.2-33.3)	ns
**Adv *vs* HMPV, n=10**	31.1 (22.9-34.7) *vs* 27.3 (22.6-33.6)	ns
**Adv *vs* HPIV, n=10**	26.7 (21.1-33.8) *vs* 26.2 (20.2-32.1)	ns
**Adv *vs* Flu viruses, n=4**	29.0 (23.5-34.9) *vs* 23.2 (18.4-27.9)	ns
**Adv *vs* *M. pneumoniae*, n=4**	33.3 (25.0-34.9) *vs* 30.8 (25.4-34.4)	ns
**Adv *vs* RSV, n=13**	33.8 (32.5-35.0) *vs* 29.1 (22.0-32.0)	p=0.0046
**Adv *vs* RV/EV, n=53**	30.0 (24.4-33.7) *vs* 30.0 (27.4-31.4)	ns
**Adv *vs* SARS-CoV-2, n=1**	24.8 (24.8-24.8) *vs* 33.5 (33.5-33.5)	ns
**HBoV *vs* Coronavirus, n=9**	34.2 (23.1-36.2) *vs* 25.9 (22.2-33.0)	ns
**HBoV *vs* HMPV, n=5**	33.5 (25.3-35.4) *vs* 31.9 (21.3-33.5)	ns
**HBoV *vs* HPIV, n=5**	29.7 (27.6-32.5) *vs* 25.2 (23.7-32.3)	ns
**HBoV *vs* Flu viruses, n=10**	32.7 (30.0-36.5) *vs* 25.0 (21.7-27.6)	ns
**HBoV *vs* *M. pneumoniae*, n=4**	31.9 (16.2-36.2) *vs* 33.9 (31.5-34.8)	ns
**HBoV *vs* RSV, n=5**	23.0 (19.5-27.7) *vs* 26.9 (21.3-29.5)	p=0.0625
**HBoV *vs* RV/EV, n=13**	28.4 (19.6-34.3) *vs* 28.0 (24.9-30.6)	ns
***B. pertussis vs* HMPV, n=4**	20.8 (17.2-30.8) *vs* 24.0 (17.1-28.7)	ns
***B. pertussis vs* HPIV, n=1**	34.1 (34.1-34.1) *vs* 23.9 (23.9-23.9)	ns
***B. pertussis vs* Flu viruses, n=2**	35.7 (35.1-36.3) *vs* 30.3 (28.7-31.8)	ns
***B. pertussis vs M. pneumoniae*, n=1**	31.6 (31.6-31.6) *vs* 23.6 (23.6-23.6)	ns
***B. pertussis vs* RV/EV, n=17**	31.0 (22.0-33.4) *vs* 29.0 (25.5-31.0)	ns
***B. pertussis vs* SARS-CoV-2, n=1**	31.0 (31.0-31.0) *vs* 35.7 (35.7-35.7)	ns
**Coronavirus *vs* HMPV, n=6**	28.9 (27.1-32.3) *vs* 32.8 (31.1-33.9)	ns
**Coronavirus *vs* HPIV, n=5**	32.9 (23.5-37.2) *vs* 26.9 (23.8-31.0)	ns
**Coronavirus *vs* Flu viruses, n=7**	30.4 (25.7-33.0) *vs* 26.9 (23.8-31.0)	ns
**Coronavirus *vs M. pneumoniae*, n=1**	22.3 (22.3-22.3) *vs* 35.2 (35.2-35.2)	ns
**Coronavirus *vs* RSV, n=14**	29.5 (25.7-32.4) *vs* 25.1 (19.6-29.5)	ns
**Coronavirus *vs* RV/EV, n=20**	24.2 (20.6-29.7) *vs* 29.6 (27.3-31.5)	ns
**HMPV *vs* HPIV, n=5**	30.4 (21.5-32.0) *vs* 24.0 (18.2-32.7)	ns
**HMPV *vs* Fu viruses, n=2**	29.3 (23.8-34.8) *vs* 26.7 (22.6-30.8)	ns
**HMPV *vs M. pneumoniae*, n=1**	19.7 (19.7-19.7) *vs* 22.0 (22.0-22.0)	ns
**HMPV *vs* RSV, n=2**	23.9 (22.4-25.3) *vs* 23.7 (23.3-24.1)	ns
**HMPV *vs* RV/EV, n=28**	22.4 (20.4-31.2) *vs* 28.9 (26.8-32.3)	p=0.0014
**HPIV *vs* Flu viruses, n=1**	33.9 (33.9-33.9) *vs* 26.7 (26.7-26.7)	ns
**HPIV *vs M. pneumoniae*, n=3**	28.6 (26.5-29.7) *vs* 30.3 (24.3-33.7)	ns
**HPIV *vs* RSV, n=5**	28.8 (24.9-32.0) *vs* 26.0 (19.6-29.5)	ns
**HPIV *vs* RV/EV, n=33**	25.2 (19.3-28.5) *vs* 30.4 (26.6-32.5)	p=0.0007
**HPIV *vs* SARS-CoV-2, n=2**	32.9 (31.3-34.4) *vs* 16.6 (14.9-18.2)	ns
**Flu viruses *vs M. pneumoniae*, n=3**	28.0 (25.1-28.9) *vs* 33.8 (32.8-36.1)	ns
**Flu viruses *vs* RSV, n=7**	23.3 (20.0-32.9) *vs* 26.5 (25.0-34.1)	ns
**Flu viruses *vs* RV/EV, n=15**	22.0 (18.9-28.6) *vs* 30.2 (27.1-32.6)	p=0.0084
***M. pneumoniae vs* RSV, n=4**	31.6 (20.2-34.7) *vs* 27.8 (22.9-31.9)	ns
***M. pneumoniae vs* RV/EV, n=15**	31.1 (28.4-34.9) *vs* 28.5 (27.2-30.8)	ns
**RSV *vs* RV/EV, n=40**	24.6 (20.3-29.6) *vs* 31.0 (28.1-32.6)	p<0.0001
**RSV *vs* SARS-CoV-2, n=6**	27.0 (21.5-34.3) *vs* 33.9 (30.9-34.5)	ns
**RV/EV *vs* SARS-CoV-2, n=4**	22.4 (19.4-31.2) *vs* 34.9 (20.5-35.5)	ns

Supplementary Table S2. Cycle threshold (Ct) values of each pathogen in respiratory combination patterns. Data are expressed as median (interquartile range, IQR). The number of positive samples (n) is reported for each combination patterns.

Ct: cycle threshold; Adv: Adenovirus; HBoV: Human Bocavirus; HMPV: Human metapneumovirus A/B; HPIV: Human parainfluenza virus; RSV: Respiratory syncytial virus A/B; RV/EV: Rhinovirus/Enterovirus; SARS-CoV-2: Severe acute respiratory syndrome coronavirus 2; ns: not significant ($p>0.05$).

Supplementary Figure S3. Hierarchical Mapping of Pathogen Dominance in sample positive for two pathogens. The heatmap displays pairwise comparisons of pathogens detected in sample positive for two pathogens. Red intensity indicates that the pathogen on the X-axis has lower Ct values (i.e. a higher viral load, negative ΔCt) than the corresponding pathogen on the Y-axis. Green intensity indicates that the pathogen on the X-axis has higher Ct values (i.e. lower viral load, positive ΔCt) than Ct values of the pathogen on the Y-axis. Orange/yellow cells indicate comparable Ct values. Blank cells indicate pathogen combination not observed.

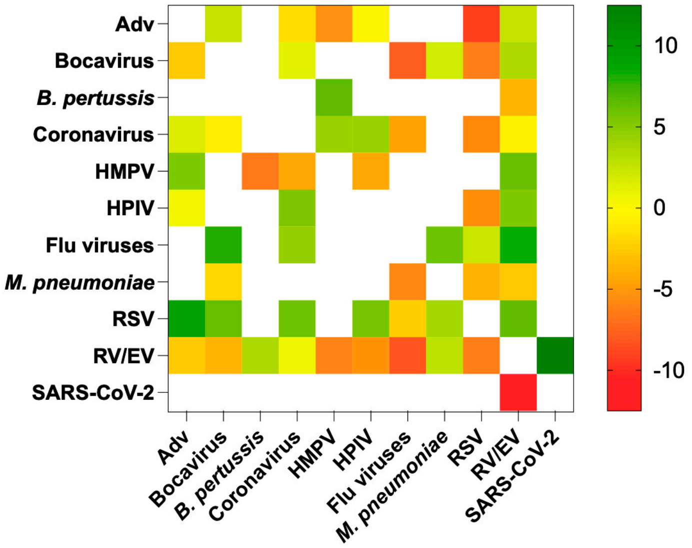

Adv: Adenovirus; HBoV: Human Bocavirus; HMPV: Human metapneumovirus A/B; HPIV: Human parainfluenza virus; RSV: Respiratory syncytial virus A/B; RV/EV: Rhinovirus/Enterovirus; SARS-CoV-2: Severe acute respiratory syndrome coronavirus 2.
